# Aging Impacts the Overall Connectivity Strength of Regions Critical for Information Transfer Among Brain Networks

**DOI:** 10.3389/fnagi.2020.592469

**Published:** 2020-10-28

**Authors:** Epifanio Bagarinao, Hirohisa Watanabe, Satoshi Maesawa, Daisuke Mori, Kazuhiro Hara, Kazuya Kawabata, Noritaka Yoneyama, Reiko Ohdake, Kazunori Imai, Michihito Masuda, Takamasa Yokoi, Aya Ogura, Toshiaki Taoka, Shuji Koyama, Hiroki C. Tanabe, Masahisa Katsuno, Toshihiko Wakabayashi, Masafumi Kuzuya, Minoru Hoshiyama, Haruo Isoda, Shinji Naganawa, Norio Ozaki, Gen Sobue

**Affiliations:** ^1^Brain and Mind Research Center, Nagoya University, Nagoya, Japan; ^2^Department of Neurology, Fujita Health University School of Medicine, Toyoake, Japan; ^3^Department of Neurology, Nagoya University Graduate School of Medicine, Nagoya, Japan; ^4^Department of Neurosurgery, Nagoya University Graduate School of Medicine, Nagoya, Japan; ^5^Department of Radiology, Nagoya University Graduate School of Medicine, Nagoya, Japan; ^6^Department of Cognitive and Psychological Sciences, Graduate School of Informatics, Nagoya University, Nagoya, Japan; ^7^Department of Community Healthcare and Geriatrics, Nagoya University Graduate School of Medicine, Nagoya, Japan; ^8^Institutes of Innovation for Future Society, Nagoya University, Nagoya, Japan; ^9^Department of Psychiatry, Nagoya University Graduate School of Medicine, Nagoya, Japan

**Keywords:** aging, connector hubs, adult lifespan, intrinsic connectivity contrast, resting state fMRI

## Abstract

Recent studies have demonstrated that connector hubs, regions considered critical for the flow of information across neural systems, are mostly involved in neurodegenerative dementia. Considering that aging can significantly affect the brain’s intrinsic connectivity, identifying aging’s impact on these regions’ overall connection strength is important to differentiate changes associated with healthy aging from neurodegenerative disorders. Using resting state functional magnetic resonance imaging data from a carefully selected cohort of 175 healthy volunteers aging from 21 to 86 years old, we computed an intrinsic connectivity contrast (ICC) metric, which quantifies a region’s overall connectivity strength, for whole brain, short-range, and long-range connections and examined age-related changes of this metric over the adult lifespan. We have identified a limited number of hub regions with ICC values that showed significant negative relationship with age. These include the medial precentral/midcingulate gyri and insula with both their short-range and long-range (and thus whole-brain) ICC values negatively associated with age, and the angular, middle frontal, and posterior cingulate gyri with their long-range ICC values mainly involved. Seed-based connectivity analyses further confirmed that these regions are connector hubs with connectivity profile that strongly overlapped with multiple large-scale brain networks. General cognitive performance was not associated with these hubs’ ICC values. These findings suggest that even healthy aging could negatively impact the efficiency of regions critical for facilitating information transfer among different functional brain networks. The extent of the regions involved, however, was limited.

## Introduction

Studies have shown that the human brain is functionally organized into several large-scale networks, which can be identified using the spontaneous low frequency fluctuations of resting state functional magnetic resonance imaging (fMRI) data (Biswal et al., 1995; Greicius et al., 2003; Beckmann et al., 2005; Seeley et al., 2007). Some of these so-called resting state networks (RSNs), or their precursors, can already be identified even in the infant brain (Fransson et al., 2007; Smyser et al., 2010). These networks continue to mature with age and are continuously transformed across the life span (Tomasi and Volkow, 2012; Betzel et al., 2014; Sala-Llonch et al., 2015; Bagarinao et al., 2019). Characterizing the changes these RSNs undergo throughout normal development and aging is important as recent studies have indicated that RSN disruptions can also be associated with psychiatric (Greicius, 2008; Menon, 2011) and neurodegenerative disorders (Greicius et al., 2004; Hacker et al., 2012; Yao et al., 2014; Dai et al., 2015; Kawabata et al., 2018; Yokoi et al., 2018; Yoneyama et al., 2018; Ogura et al., 2019).

A number of brain regions called cortical hubs, characterized by numerous strong interconnections with several other regions, are considered critical for the flow of information within and between brain networks (Sporns et al., 2007). Prominent hub regions include the posterior cingulate, lateral temporal, lateral parietal, and medial/lateral prefrontal, many of which overlap with regions of the default mode network (Buckner et al., 2009). Given this critical role, hub regions are generally implicated in the anatomy of many disorders (Crossley et al., 2014). Its dysfunctions are also associated with behavioral and cognitive impairments in several neurological and psychiatric disorders (Buckner et al., 2009; van den Heuvel et al., 2013; van den Heuvel and Sporns, 2013; Dai et al., 2015) and could also provide novel insight into the pathomechanism of cognitive decline (Ogura et al., 2019).

Since the human brain processes information not only within adjacent areas but also from distant projections, recent studies have also investigated hub profiles in a distance-dependent manner (Sepulcre et al., 2010; Alexander-Bloch et al., 2013; Liang et al., 2013; Dai et al., 2015). Some hub regions were found to have high preference to local connectivity involving regions mostly in primary and secondary cortices (motor, somatosensory, auditory, visual, etc.), or long distance connectivity involving heteromodal regions in lateral parieto-temporal and frontal cortices, or both local and long distance connectivity involving regions which overlap with the default mode network (Sepulcre et al., 2010). By taking into account the distance of functional connections, certain vulnerabilities to distance-dependent connectivity changes can be identified. There are also differences in metabolic demands for long-range brain hubs, which require more energy, as compared to short-range hubs, making the former more vulnerable than the latter (Bullmore and Sporns, 2012).

Considering that aging can significantly affect the brain’s intrinsic connectivity, characterizing age-related changes in the overall connectivity strength of hub regions is important in order to differentiate changes associated with healthy aging from that due to neurodegenerative disorders. In this study, we examined aging’s impact on the overall connection strength of hub regions. For this, we used resting state fMRI data to construct an intrinsic connectivity contrast (ICC) map (Martuzzi et al., 2011), which reflects each voxel’s overall connectivity strength. Using a carefully selected cohort of healthy volunteers with age ranging from 21 to 86 years old, we first investigated the characteristics of the ICC map in a subgroup of young participants, and then examined how the ICC values change with age over the adult lifespan. In addition, we also examined whether some of the changes are distance-dependent (Euclidean) following earlier studies highlighting differences in the connectivity profile of hubs with respect to the distance of their connection. Finally, we performed seed-based connectivity analyses using as seed regions those with ICC values that showed significant association with age to identify the regions’ connections to several well-known RSNs.

## Materials and Methods

### Participants

Resting state fMRI data from 175 healthy volunteers (85 males/90 females) ranging in age from 21 to 86 years were included in the analysis. The included participants were carefully selected from those enrolled in our ongoing Brain and Mind Research Center Aging Cohort Study (Bagarinao et al., 2018, 2019) using the following exclusion criteria: 1) inability to complete the Japanese version of Addenbrooke’s Cognitive Examination-Revised (ACE-R) assessment, 2) presence of structural abnormalities (e.g., asymptomatic cerebral infarction, benign brain tumor, white matter abnormalities, etc.) in structural MRI as identified by two Japanese board-certified neurologists (HW, KH) and a neurosurgeon (SM), 3) Mini-Mental State Examination (MMSE) score less than 26 or ACE-R total score less than 89, 4) head motion in resting state fMRI data with mean frame-wise displacement (FD) (Power et al., 2012) values greater than 0.2 mm, and 5) incomplete imaging data. Although MMSE score was also assessed, this score was only used for additional screening of the participants. The participants’ characteristics are summarized in Table 1. All participants gave written informed consent before joining the study, which was approved by the Ethics Committee of Nagoya University Graduate School of Medicine and conformed to the Ethical Guidelines for Medical and Health Research Involving Human Subjects as endorsed by the Japanese Government.

**TABLE 1:** Participants’ characteristics.

Age Range	Count	M	F	Mean ACE-R (SD) score					
				Total	Attn	Mem	Flncy	Lang	Visu
20–29	39	25	14	96.5 (1.89)	17.9 (0.22)	24.2 (1.11)	13.8 (0.48)	24.9 (1.30)	15.7 (0.68)
30–39	21	10	11	97.9 (2.49)	18.0 (0.00)	25.0 (1.24)	13.4 (1.40)	25.4 (0.81)	16.0 (0.00)
40–49	19	11	8	96.4 (3.13)	17.9 (0.23)	24.3 (1.88)	13.4 (1.07)	25.0 (1.11)	15.7 (0.65)
50–59	34	12	22	97.4 (1.54)	17.9 (0.24)	24.6 (1.35)	13.7 (0.57)	25.4 (0.73)	15.8 (0.50)
60–69	35	14	21	96.1 (2.41)	17.9 (0.40)	23.7 (2.15)	13.8 (0.53)	25.2 (0.83)	15.5 (0.92)
70–79	26	13	13	94.8 (3.06)	17.8 (0.40)	23.6 (1.94)	13.1 (1.14)	24.8 (1.20)	15.5 (0.86)
80–89	1	0	1	91.0 (–)	17.0 (–)	20.0 (–)	14.0 (–)	25.0 (–)	15.0 (–)
Total	175	85	90						

*SD, standard deviation; M, male; F, female; ACE-R, Addenbrooke’s Cognitive Examination-Revised; Attn, attention; Mem, memory; Flncy, fluency; Lang, language; Visu, visuospatial.*

### Magnetic Resonance Imaging (MRI) Data

All participants underwent MRI scanning at the Brain and Mind Research Center using a Siemens Magnetom Verio (Siemens, Erlangen, Germany) 3.0T scanner with a 32-channel head coil. For each participant, a high resolution T1-weighted (T1w) image and resting state fMRI data were acquired. The T1w image was acquired using a 3D MPRAGE (Magnetization Prepared Rapid Acquisition Gradient Echo, Siemens) pulse sequence (Mugler and Brookeman, 1990) with the following parameters: repetition time (TR)/MPRAGE repetition time = 7.4/2500 ms, echo time (TE) = 2.48 ms, inversion time (TI) = 900 ms, flip angle (FA) = 8 degrees, field of view (FOV) = 256 mm, 256 × 256 matrix dimension, 192 sagittal slices with 1-mm thickness, in-plane voxel resolution of 1.0 × 1.0 mm^2^, and total scan time of 5 min and 49 s. For the resting state fMRI data, an ascending gradient echo (GE) echo planar imaging (EPI) pulse sequence was used with the following imaging parameters: TR = 2.5 s, TE = 30 ms, FOV = 192 mm, 64 × 64 matrix dimension, FA = 80 degrees, 39 transverse slices with a 0.5-mm inter-slice interval and 3-mm slice thickness, and total scan time of 8 min and 15 s. During resting state fMRI scan, participants were instructed to close their eyes but not to fall asleep.

### Image Preprocessing

All images were preprocessed using Statistical Parametric Mapping (SPM12, Wellcome Trust Center for Neuroimaging, London, United Kingdom) running on Matlab (R2016b, MathWorks, Natick, MA, United States). Using SPM12’s segmentation approach (Ashburner and Friston, 2005), the T1w images were segmented into component images including gray matter (GM), white matter (WM), cerebrospinal fluid (CSF), and other non-brain tissue components. Bias-corrected T1w images and the transformation information from subject space to the Montreal Neuroimaging Institute (MNI) space were also obtained. For each resting state fMRI data, the first five volumes in the series were removed. Based on our previous work, this number is already sufficient to account for the initial BOLD signal instability. The remaining images were then slice-time corrected relative to the middle slice (slice 20), and then realigned to the mean image, computed after initially realigning the images relative to the first image. The mean image, together with the realigned images, were then co-registered to the bias-corrected T1w image, normalized to the MNI space using the transformation information obtained during segmentation, resampled to an isotropic 2 × 2 × 2 mm^3^ voxel resolution, and smoothed using an isotropic 8-mm full-width-at-half-maximum 3-dimensional Gaussian filter. The preprocessed images were then corrected for head motion and contribution from other nuisance signals. In particular, we regressed out 24 motion-related signals given by [*R*_*t*_, *R*_*t*_^2^, *R*_*t* – 1_, and *R*_*t* – 1_^2^], where *R*_*t*_ = [*x*_*t*_, *y*_*t*_, *z*_*t*_, α*_*t*_*, β*_*t*_*, γ*_*t*_*] represents the estimated motion parameters (*x*, *y*, and *z* for translations and α, β, and γ for rotations about *x*, *y*, and *z*, respectively) at time *t*. Mean signals from spherical regions of interest (radius = 4 mm) within the CSF and WM, the global signal, as well as derivatives of these signals were also removed. Finally, a bandpass filter within the frequency range from 0.01 to 0.1 Hz was also applied. These additional image preprocessing steps were performed using functions available in Matlab.

### Intrinsic Connectivity Contrast

From the preprocessed functional images, we computed the voxel-wise ICC (Martuzzi et al., 2011) using Matlab. Specifically, we used the form of the ICC that computes the power (ICC-p_th_) with the threshold set to 0 and computed using the following formula:

$$\begin{array}{c} ICC\left(i\right)=\frac{1}{n}\sum_{j\neq i}^{n}r{\left(i,j\right)}^{2} \end{array}$$

where *r(i,j)* is the connectivity value between voxels *i* and *j* and *n* is the number of voxels. To exclude unnecessary voxels, we limit our analysis to only include GM-relevant voxels. For this, we generated a GM mask by applying a threshold value of 0.2 to SPM12’s GM tissue probability map. Voxels with values exceeding this threshold were included in the GM mask. The ICC values of all voxels within the mask were then computed resulting in a map of ICC values. Finally, the ICC values were standardized by subtracting the mean ICC computed from all voxels in the image, and dividing the difference by the standard deviation. This conversion to *z*-score does not affect the topography of the individual maps but enables the maps to be averaged and compared across participants (Buckner et al., 2009).

**FIGURE 1:**
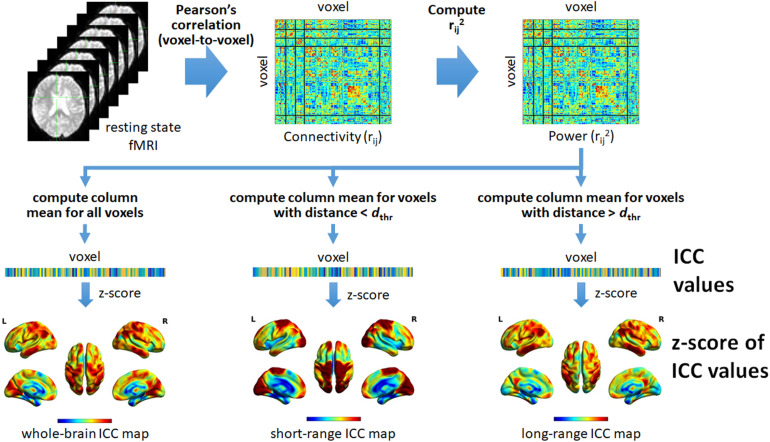
Schematic illustration of the different steps to compute the intrinsic connectivity contrast (ICC) values from resting state fMRI data. Using the preprocessed data, we computed Pearson’s correlation (*r*_*ij*_) between pairs of voxels within the brain resulting in a whole-brain connectivity matrix quantifying voxel-to-voxel connectivity. The square of the value of each element in the connectivity matrix was then computed in order to obtain the form of the ICC that computes the power (*r*_*ij*_^2^). The resulting squared connectivity matrix or “power” matrix was then used to generate the whole-brain, short-range, and long-range ICC maps. For the whole-brain ICC map, the column-wise mean of the power matrix was computed (excluding self-connection). The resulting mean values were then converted to z-scores to form the map. For the short-range ICC map, only voxels with (Euclidean) distance less than a given threshold, denoted as *d*_thr_, relative to the reference voxel (voxel *i* for column *i*) were included in computing the mean values. On the other hand, for the long-range ICC map, only voxels with distance greater than *d*_thr_ were included. For both, the computed mean values were also converted to z-scores to form the corresponding maps. L, left; R, right.

Distance-dependent ICC values were also computed. For the short-range ICC values, only voxels within distance *d*_thr_ (Euclidean) from the reference voxel were included in the computation. On the other hand, for long-range ICC values, only voxels with distance greater than *d*_thr_ were included. In this study, we used *d*_thr_ = 75 mm to be consistent with the value used in previous studies (Achard et al., 2006; He et al., 2007). The different steps in computing the different ICC maps are summarized in Figure 1.

### Statistical Analyses

Although hub regions identified using the network metric called degree are known (Buckner et al., 2009), hub regions identified using ICC are yet to be characterized. Given this, we first profiled the spatial distribution of ICC values across the brain for whole-brain, short-range, and long-range connections. For this analysis, we used data from a subset of participants with age less than 40 years old (*N* = 60). We performed one-sample *t*-tests of the ICC maps for the whole brain, long-range, and short-range ICC values using SPM12. To identify voxels with the most significant ICC values, we applied a more stringent *p*-value threshold equal to 0.05 corrected for multiple comparisons using a family-wise error (FWE) rate and cluster size equal to or greater than 25 voxels to the resulting statistical maps. We note that this analysis is primarily aimed to establish the spatial profile of the ICC metric across the whole brain, rather than to make comparisons across different age groups. To examine age-related changes in ICC values, which is our primary goal, we used regression analysis as described next.

We examined the relationship between ICC values and age and cognitive performance using data from all participants. Since all participants were cognitively normal, the variability of several ACE-R sub-scores were very limited (Table 1), preventing us to investigate associations at specific cognitive subdomains. We therefore used the ACE-R total score as the metric representing general cognitive performance. We used a linear regression model with age and ACE-R total score as regressors. We also included sex and the mean FD values estimated from the realignment parameters (Power et al., 2012) as regressors of no interest to account for potential sex differences and the residual effects of head motion. Regression coefficients were estimated using SPM12. Generated statistical maps were thresholded using a *p*-value equal to 0.05 corrected for multiple comparisons using FWE at the cluster level (FWEc) with a cluster defining threshold (CDT) set at *p* = 0.001.

### Seed-Based Connectivity Analysis and RSN Overlap Ratio

We also performed additional seed-based connectivity analyses using regions with ICC values that showed significant relationship with age to quantify the regions’ connectivity relative to well-known RSNs. This would be useful in identifying connector hubs from non-connector hubs as the former will be significantly connected to not just one but multiple RSNs. Spherical seed regions-of-interest (ROIs) with centers located at the MNI coordinates of the significant clusters’ peak locations and radius equal to 4 mm were used. For each seed ROI, its mean time series was extracted and correlated to that of all voxels within the brain (whole-brain connections) to generate its corresponding functional connectivity map. Using the generated connectivity maps from participants in the young adult subgroup, a one-sample *t*-test was then performed. The resulting statistical map was corrected for multiple comparisons using FWEc *p* < 0.05 with a CDT set at *p* = 0.001 to obtain the group-level functional connectivity map of the given ROI. To quantify the seed ROI’s connectivity with several RSNs, we estimated the spatial overlap of the thresholded statistical map with well-known RSNs. For this, we used the 14 RSN templates from Shirer et al. (2012) as reference and computed an RSN overlap ratio *R* (Bagarinao et al., 2020) using the following equation:

$$\begin{array}{c} R=\frac{N_{overlap}}{N_{RSN}}, \end{array}$$

where *N*_*overlap*_ is the number of voxels in the RSN template that overlapped with the FWEc-corrected statistical map of the ROI, and *N*_*RSN*_ is the total number of voxels in the RSN template. An overlap ratio of 1 means that the RSN template is fully within the corrected connectivity map of the given seed ROI (*N*_*overlap*_ = *N*_*RSN*_), whereas a value of 0 means no overlap at all (*N*_*overlap*_ = 0). We used the RSN overlap ratio to identify the different RSNs where the seed ROI has significant functional connection.

## Results

### ICC Characteristics in Young Adult Participants

**FIGURE 2:**
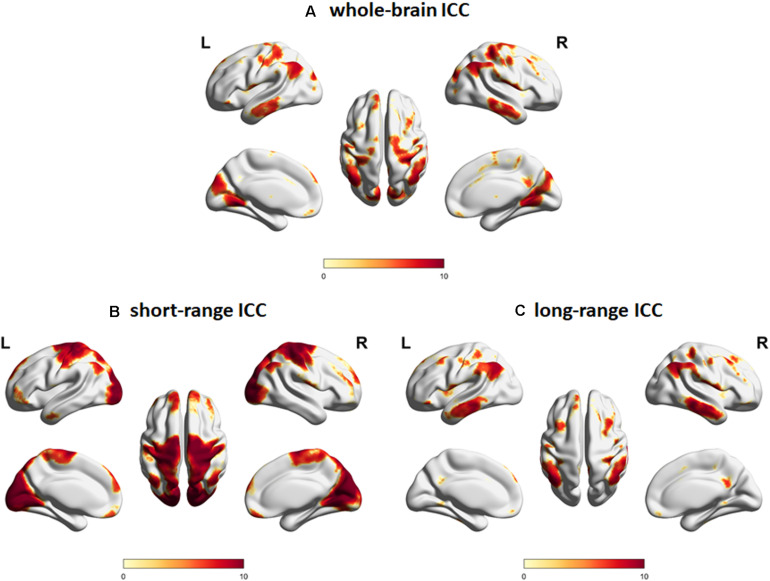
Intrinsic connectivity contrast (ICC) maps for **(A)** whole-brain connections, **(B)** short-range connections, and **(C)** long-range connections constructed using resting state fMRI data from the young adult subgroup (age <40 years old, *N* = 60). Regions with significant (FWEc *p* < 0.05, CDT *p* = 0.001) ICC values are indicated. Color map encodes *t*-values mapped on the cortical surface using BrainNet Viewer (Xia et al., 2013). L, left; R, right.

We first characterized the spatial distribution of the ICC maps in a subgroup of young adult participants. Whole-brain ICC maps identified hub regions showing significant ICC values in the bilateral middle frontal gyrus, angular gyrus, middle temporal gyrus, precuneus, medial superior frontal gyrus, lateral orbital gyrus, lingual gyrus, and central operculum, among others (Figure 2A). For the distance-dependent ICC maps, cortical hubs characterized by significant short-range ICC values were observed in regions associated with primary processing networks (visual and sensorimotor) as well as in bilateral medial superior frontal gyrus, lateral orbital gyrus/orbital part of the inferior frontal gyrus, lateral parietal, right central operculum, and cerebellum (Figure 2B). In contrast, cortical hubs characterized by significant long-range ICC values were observed in regions associated with the core neurocognitive networks (default mode, salience, and executive control) such as the bilateral middle frontal gyrus, angular/supramarginal gyrus, inferior/middle temporal gyrus, precuneus/posterior cingulate gyrus, and midcingulate gyrus, among others (Figure 2C). MNI coordinates of the peak locations and sizes of the significant clusters are given in Supplementary Tables S1–S3 for whole-brain, short-range, and long-range ICC values, respectively.

### Relationship Between ICC Values and Age

**FIGURE 3:**
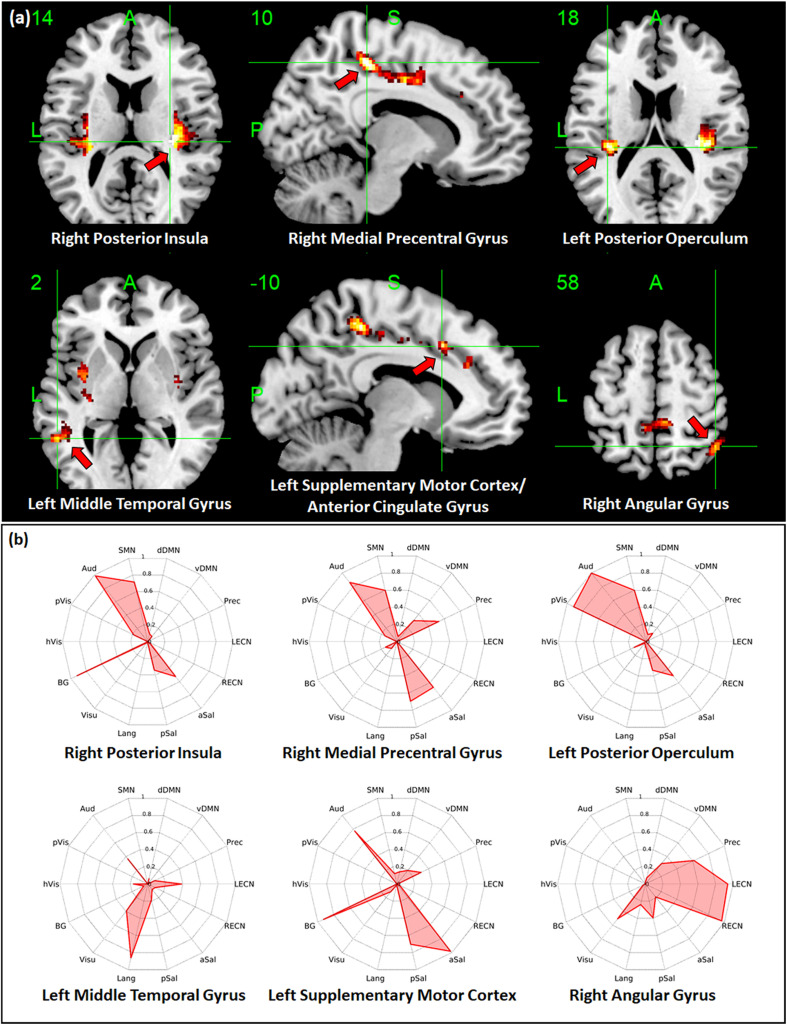
Relationship between whole-brain intrinsic connectivity contrast (ICC) values and age. **(a)** Regions with whole-brain ICC values that showed significant (FWEc *p* < 0.05, CDT *p* = 0.001) negative linear relationship with age. **(b)** Spider plots of the overlap ratio quantifying the connection between the 14 canonical resting state networks and the regions shown in **(a)**. dDMN, dorsal default mode network; vDMN, ventral default mode network; Prec, precuneus network; LECN, left executive control network; RECN, right executive control network; aSal, anterior salience network; pSal, posterior salience network; Lang, language network; Visu, visuospatial (dorsal attention) network; BG, basal ganglia network; hVis, high visual network; pVis, primary visual network; Aud, auditory network; SMN, sensorimotor network; R, right; L, left.

**FIGURE 4:**
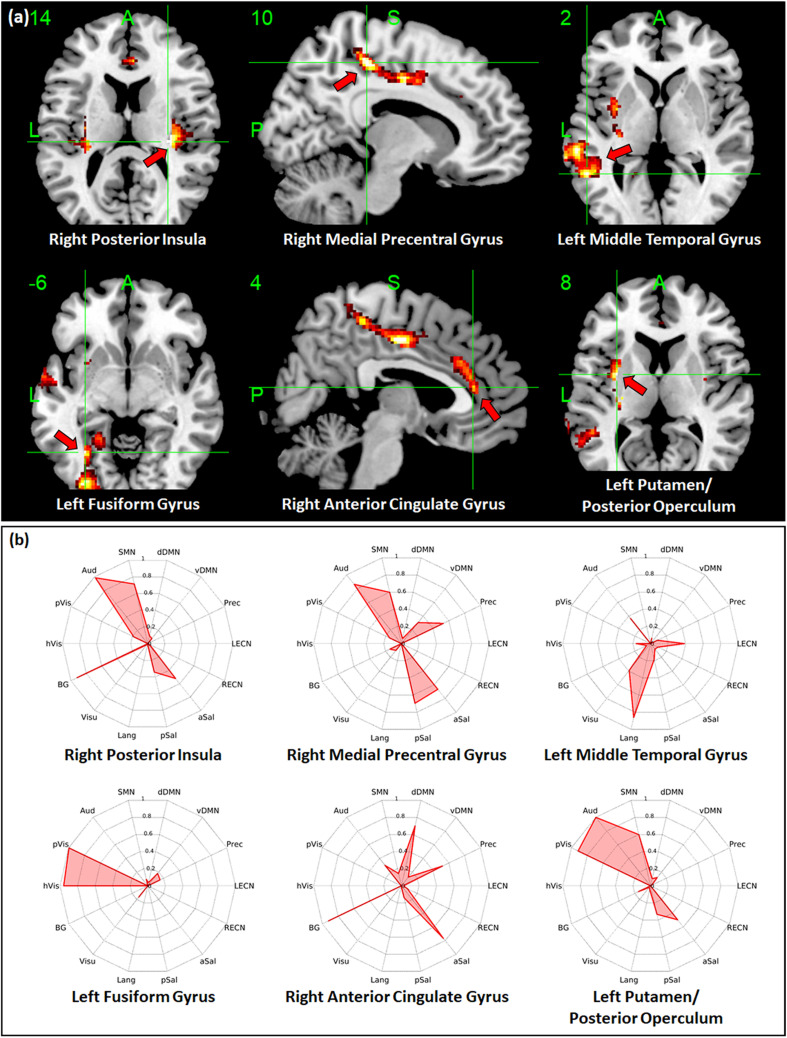
Relationship between short-range intrinsic connectivity contrast (ICC) values and age. **(a)** Regions with short-range ICC values that showed significant (FWEc *p* < 0.05, CDT *p* = 0.001) negative linear relationship with age. **(b)** Spider plots of the overlap ratio quantifying the connection between the 14 canonical resting state networks and the regions shown in **(a)**. dDMN, dorsal default mode network; vDMN, ventral default mode network; Prec, precuneus network; LECN, left executive control network; RECN, right executive control network; aSal, anterior salience network; pSal, posterior salience network; Lang, language network; Visu, visuospatial (dorsal attention) network; BG, basal ganglia network; hVis, high visual network; pVis, primary visual network; Aud, auditory network; SMN, sensorimotor network; R, right; L, left.

**FIGURE 5:**
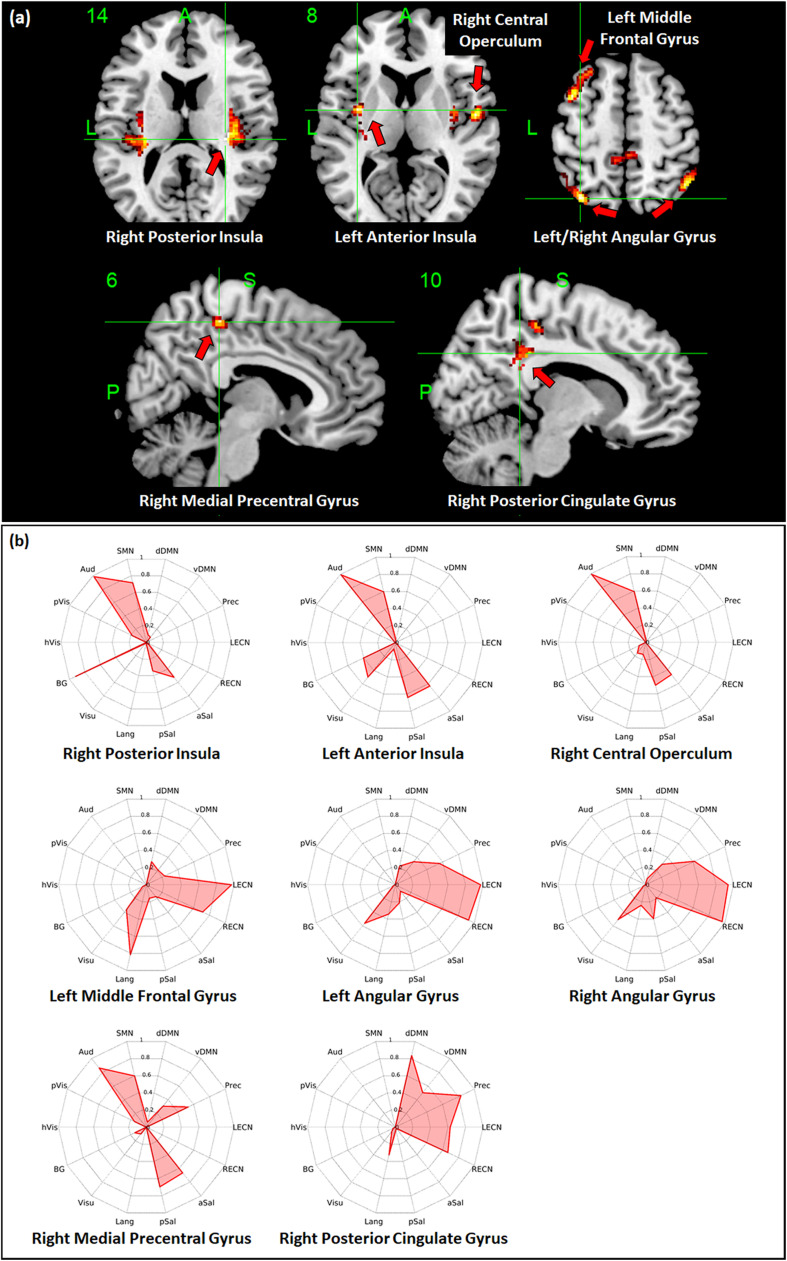
Relationship between long-range intrinsic connectivity contrast (ICC) values and age. **(a)** Regions with long-range ICC values that showed significant (FWEc *p* < 0.05, CDT *p* = 0.001) negative linear relationship with age. **(b)** Spider plots of the overlap ratio quantifying the connection between the 14 canonical resting state networks and the regions shown in **(a)**. dDMN, dorsal default mode network; vDMN, ventral default mode network; Prec, precuneus network; LECN, left executive control network; RECN, right executive control network; aSal, anterior salience network; pSal, posterior salience network; Lang, language network; Visu, visuospatial (dorsal attention) network; BG, basal ganglia network; hVis, high visual network; pVis, primary visual network; Aud, auditory network; SMN, sensorimotor network; R, right; L, left.

Regions with whole-brain ICC values that showed significant negative linear relationship with age included the bilateral insula, bilateral medial segment of the precentral gyrus/midcingulate, left middle temporal gyrus, left anterior cingulate gyrus, and right angular gyrus (Figure 3a). Age-related changes in whole-brain ICC values in the bilateral insula and the medial segment of the precentral gyrus/midcingulate gyrus were influenced by both the short-range and long-range ICC values, which also showed significant negative relationship with age (Figures 4a, 5a). On the other hand, age-related changes in whole-brain ICC values in the left anterior cingulate gyrus and middle temporal gyrus were mostly influenced by the short-range ICC values (Figure 4a), whereas that in the right angular gyrus by the long-range ICC values (Figure 5a). A region in the left fusiform gyrus has only short-range ICC values that showed significant negative relationship with age (Figure 4a). In contrast, regions in the right central operculum, bilateral angular gyrus, left middle frontal gyrus, and posterior cingulate gyrus have only long-range ICC values that showed significant negative linear relationship with age (Figure 5a).

Regions in the bilateral caudate and right cerebellum have whole-brain, short-range, and long-range ICC values that showed significant positive relationship with age. Other regions with ICC values exhibiting positive association with age included the right thalamus and left hippocampus for whole-brain ICC values, the right lingual gyrus for both whole-brain and long-range ICC values, and the right planum polare/temporal pole for both whole-brain and short-range ICC values. Peak locations in MNI coordinates and sizes of the significant clusters are given in Tables 2–4 for whole-brain, short-range, and long-range ICC values, respectively.

**TABLE 2:** Regions showing significant (FWEc *p* < 0.05, CDT *p* = 0.001) relationship between whole-brain ICC values and age and ACE-R total score.

Contrast	*X*	*Y*	*Z*	*z*-Value	Cluster size	Area	Other within cluster peaks
Age (+)	−10	14	2	6.61	436	L Cau	
	14	16	6	6.17	267	R Cau	
	14	−34	20	6.1	149	R ThP	
	34	−34	−40	5.47	371	R Cer	
	4	−98	−4	4.86	182	R LiG	R OCP, L OCP
	44	−10	−10	4.77	179	R PP	R TMP
	−18	−44	16	4.63	249	L Hip	
	34	18	−42	4.48	168	R TMP	
Age (−)	30	−24	14	5.99	349	R PIns	R AIns
	10	−32	52	5.48	955	R MPrG	R MCgG, L MPrG
	−36	−28	18	5.02	446	L PO	L AIns, L PIns
	−10	16	40	4.62	180	L SMC	L ACgG, R ACgG
	−54	−48	2	4.4	136	L MTG	L STG
	44	−54	58	4.34	128	R AnG	R SPL
ACE-R (+)	−34	−74	−16	4.47	380	L OFuG	L Calc
	18	−76	6	4.35	744	R Calc	R OFuG, R LiG
	−18	−86	26	4.29	425	L SOG	L OCP
	−30	−24	62	4.08	220	L PrG	L PoG
	−20	−54	0	3.9	121	L LiG	
	−10	−72	−56	4.21	218	L Cer	

*Cau, caudate; ThP, thalamus proper; Cer, cerebellum; LiG, lingual gyrus; OCP, occipital pole; PP, planum polare; TMP, temporal pole; Hip, hippocampus; PIns, posterior insula; AIns, anterior insula; MPrG, medial precentral gyrus; MCgG, midcingulate gyrus; PO, posterior operculum; SMC, supplementary motor cortex; ACgG, anterior cingulate gyrus; MTG, middle temporal gyrus; STG, superior temporal gyrus; AnG, angular gyrus; SPL, superior parietal lobule; OFuG, occipital fusiform gyrus; Calc, calcarine; SOG, superior occipital gyrus; PrG, precentral gyrus; PoG, postcentral gyrus; ACE-R, Addenbrooke’s Cognitive Examination-Revised; L, left; R, right.*

**TABLE 3:** Regions showing significant (FWEc *p* < 0.05, CDT *p* = 0.001) relationship between short-range ICC values and age and ACE-R total score.

Contrast	*X*	*Y*	*Z*	*z*-Value	Cluster size	Area	Other within cluster peaks
Age (+)	−10	14	2	6.49	467	L Cau	
	14	16	6	6.15	382	R Cau	
	−46	−8	−8	5.42	171	L PP	
	36	−34	−38	5.24	371	R Cer	R FuG
	46	−6	−10	4.97	378	R PP	R TMP
	34	18	−44	4.95	204	R TMP	
Age (−)	30	−24	14	5.77	219	R PIns	R Pu
	10	−32	52	5.41	1046	R MPrG	R MCgG, L MCgG
	−32	−58	−6	4.96	625	L FuG	L IOG, L OFuG
	−54	−48	2	4.93	675	L MTG	L STG
	−32	0	8	4.8	384	L Pu	L PO, L PIns
	4	36	14	4.26	408	R ACgG	L ACgG, L SMC
ACE-R (+)	−20	−86	28	4.45	1,426	L SOG	R Calc
	−18	−86	−16	4	226	L OFuG	
	−20	−54	0	3.86	217	L LiG	L Calc
ACE-R (−)	22	2	60	4.76	174	R SFG	

*Cau, caudate; PP, planum polare; Cer, cerebellum; FuG, fusiform gyrus; TMP, temporal pole; PIns, posterior insula; Pu, putamen; MPrG, medial precentral gyrus; MCgG, midcingulate gyrus; IOG, inferior occipital gyrus; OFuG, occipital fusiform gyrus; MTG, middle temporal gyrus; STG, superior temporal gyrus; PO, posterior operculum; ACgG, anterior cingulate gyrus; SMC, supplementary motor cortex; SOG, superior occipital gyrus; Calc, calcarine; LiG, lingual gyrus; SFG, superior frontal gyrus; ACE-R, Addenbrooke’s Cognitive Examination-Revised; L, left; R, right.*

**TABLE 4:** Regions showing significant (FWEc *p* < 0.05, CDT *p* = 0.001) relationship between long-range ICC values and age and ACE-R total score.

Contrast	*X*	*Y*	*Z*	*z*-Value	Cluster size	Area	Other within cluster peaks
Age (+)	−10	14	2	6.31	476	L Cau	
	14	16	6	5.99	230	R Cau	
	34	−34	−40	5.03	165	R Cer	R FuG
	4	−98	−4	4.78	309	R LiG	R OCP, L LiG
Age (−)	30	−24	14	5.51	339	R PIns	R AIns, R PO
	52	−6	6	5.04	146	R CO	
	−36	−2	8	4.77	330	L AIns	L PO, L PIns
	−34	−68	56	4.77	260	L AnG	
	−40	8	56	4.75	233	L MFG	
	6	−34	54	4.7	220	R MPrG	L MPrG
	44	−58	56	4.63	215	R AnG	
	16	−42	34	4.59	112	R PCgG	
ACE-R (+)	−34	−74	−16	4.61	124	L OFuG	L IOG
	30	−66	−10	4.33	369	R OFuG	R LiG
	−30	−22	62	4.29	308	L PrG	L PoG
ACE-R (−)	−10	−56	−64	4.31	351	L Cer	

*Cau, caudate; Cer, cerebellum; FuG, fusiform gyrus; LiG, lingual gyrus; OCP, occipital pole; PIns, posterior insula; AIns, anterior insula; PO, posterior operculum; CO, central operculum; AnG, angular gyrus; MFG, middle frontal gyrus; MPrG, medial precentral gyrus; PCgG, posterior cingulate gyrus; OFuG, occipital fusiform gyrus; IOG, inferior occipital gyrus; PrG, precentral gyrus; PoG, postcentral gyrus; ACE-R, Addenbrooke’s Cognitive Examination-Revised; L, left; R, right.*

### Overlap of Seed-Based Connectivity Maps With Large-Scale RSNs

To quantify the connectivity between regions with ICC values showing significant negative relationship with age and large-scale functional networks, we computed the overlap ratio between the estimated connectivity map of each region and 14 well-known RSN templates. Spider plots of the overlap ratio for the different seed ROIs showing age-related changes in whole-brain, short-range, and long-range ICC values are shown in Figures 3b, 4b, and 5b, respectively. From these figures, it is evident that the identified ROIs have connectivity profiles that overlapped with not just one but several RSNs indicating that these regions are mostly connector hub regions. Regions with whole-brain, short-range, and long-range ICC values that showed significant negative relationship with age (right posterior insula, right medial precentral gyrus, and left posterior operculum) had strong overlap with primary processing networks (sensorimotor, auditory, and visual) and salience network (Figure 3b). In contrast, regions with long-range ICC values that showed significant negative relationship with age (bilateral angular gyrus, left middle frontal gyrus, and right posterior cingulate gyrus) were strongly connected with control (visuospatial, executive control, and salience) and default mode networks.

### Relationship Between ICC Values and ACE-R Total Score

From Tables 2–4, one can see that some regions in the visual cortex (occipital fusiform gyrus, calcarine, and superior occipital gyrus), sensorimotor cortex (left precentral and postcentral gyri), and cerebellum have whole-brain, short-range, and long-range ICC values that positively correlated with ACE-R total score. All of the regions do not overlap with those showing significant negative relationship with age except the one in the left lingual gyrus with short-range ICC values that showed significant positive relationship with ACE-R total score and negative relationship with age.

## Discussion

Using resting state fMRI, we estimated ICC values at the voxel-level. We first characterized whole-brain as well as distance-dependent ICC maps in a subgroup of young adult participants, and then examined how the ICC values change with age over the adult lifespan. Our results showed that regions with significantly higher ICC values were generally consistent with previously identified hubs (Buckner et al., 2009; Sepulcre et al., 2010), thus validating the use of the ICC metric as an effective measure to characterize hub regions. Furthermore, we have identified a limited number of regions with ICC values that showed significant negative relationship with age. These include regions in the medial precentral gyrus and insula with whole-brain as well as distance-dependent ICC values that showed significant negative relationship with age. Seed-based connectivity analyses have indicated that these regions have connectivity maps that strongly overlapped with both primary processing and salience networks. Regions in the angular gyrus, middle frontal gyrus, and posterior cingulate gyrus have also ICC values, primarily with their long-range connections, that exhibited significant negative relationship with age. These regions’ connectivity maps strongly overlapped with control (visuospatial, salience, and executive control) and default mode networks. Taken together, these findings suggest that even healthy aging could negatively impact the overall connectivity strength of connector hubs, regions critical for the exchange of information across different brain networks. The extent, however, was limited.

Several studies have investigated the effect of normal aging in the connectivity of large-scale brain networks and the organization of functional brain networks across the lifespan (Andrews-Hanna et al., 2007; Jones et al., 2011; Tomasi and Volkow, 2012; Betzel et al., 2014; Bagarinao et al., 2019). Age-related connectivity changes were found to be widespread across many networks. Commonly observed changes included decreases in connectivity within large-scale functional networks, whereas connectivity between networks tended to increase (Betzel et al., 2014; Bagarinao et al., 2019). Using graph-theoretic framework, other studies have also indicated greater functional network reorganization in the brain with increasing age reflected in age-related alterations in network metrics such as modularity, local efficiency and global efficiency (Achard and Bullmore, 2007; Chan et al., 2014; Song et al., 2014; Geerligs et al., 2015). Our own study (Bagarinao et al., 2019) has also shown that the aging brain is characterized by decreasing path length, increasing global efficiency and network degree, and decreasing betweenness, indicating a tendency of the aging brain to re-organize toward a more integrated functional network with, probably, a more random topology (Bullmore and Sporns, 2012). Overall, widespread connectivity alterations and functional network re-organization have been consistently observed with aging.

In this study, we focused on aging’s effect on a specialized set of regions called hubs, which still remains largely unexplored. For this, we used ICC, a voxel-level metric, which enabled us to account for both the presence of connections among voxels as well as the strength of these connections (Martuzzi et al., 2011). Since ICC can be computed at the voxel level, this approach also avoided parcelation-related biases on the estimation of metrics characterizing brain networks (Smith et al., 2011). With ICC, we have also avoided the problem of identifying the appropriate connectivity threshold to use in order to define the network since ICC can be estimated without applying any threshold. Moreover, since aging could affect connectivity strength in a continuous manner, estimating the overall connectivity strength as quantified by the ICC values, rather than just the number of connections, would be more appropriate. As demonstrated in Figure 2, regions with significant ICC values were generally consistent with previously identified hubs (Buckner et al., 2009; Sepulcre et al., 2010), thus validating the efficacy of this metric to characterize hub regions and with the mentioned additional advantages.

A related work by Hampson et al. (2012) had investigated age-related changes in the intrinsic connectivity patterns in young and middle aged adults using voxel-level degree measure. They found increases in network degree with age in the paralimbic cortical and subcortical areas as well as decreases in cortical areas including that in the visual and default mode networks. Consistent with their findings, we found similar increases in whole-brain ICC values with age in the caudate, thalamus, cerebellum, hippocampus, and temporal pole. Moreover, we also found similar decreases in ICC values in the lateral parietal (whole-brain and long-range connections), posterior cingulate (long-range connections) and medial prefrontal (short-range connections). Although there were also differences, these could be driven by differences in the used metric, the range of age of the study participants, and the number of participants, among other factors.

Characterizing changes in hub regions during normal aging is important since these regions are pivotal in the flow of information within and between networks (Sporns et al., 2007) and are generally implicated in the anatomy of many neurological disorders (Crossley et al., 2014). For instance, Alzheimer’s disease (AD) has been shown to selectively target highly connected hub regions in the medial and lateral prefrontal cortices, insula, and thalamus (Dai et al., 2015). The impairment of hub regions was also shown to be connectivity distance-dependent with most disruptions occurring in the long-range connections. Compared to these findings, our results showed that the extent of hub regions involved in aging is somewhat limited. Moreover, most of the regions involved in aging generally differed from that reported in AD, although there were few similarities (left insula, anterior cingulate gyrus, and inferior parietal lobule). Considering also that previous aging studies have reported widespread age-related connectivity changes affecting large-scale brain networks (Betzel et al., 2014; Bagarinao et al., 2019), our findings seemed to suggest that the aging process may mainly affect peripheral non-hub regions, whereas neurodegenerative disorders may predominantly involve connectivity alterations of critical hub regions (Buckner et al., 2009; Dai et al., 2015).

By differentiating the contribution of the different connections in terms of (Euclidean) distance, we were also able to identify differences in hub profiles using distance-dependent ICC values (Figure 2). Similar to a previous study that used network degree to categorize hubs (Sepulcre et al., 2010), we also found that some hub regions were predominantly characterized by their short-range connections (e.g., primary processing systems), whereas others with their long-range connections (e.g., posterior cingulate gyrus and inferior/middle temporal gyrus). In addition, a number of regions such as in the lateral parietal, orbitofrontal gyrus, middle frontal gyrus, and medial superior frontal gyrus showed hub characteristics in both their short-range and long-range connections. Identified hubs with predominant short-range connections are typically located within or near primary sensory or motor areas and may therefore be involved in local information processing. On the other hand, those with predominant long-range connections are regions that have been associated with higher cognitive functions, which require information integration across distributed sources that could be readily achieved using long-range connections. Hub regions that exhibited both short-range and long-range connections are also known association regions, thus the predominant long-range connections are consistent with their known functions. The role of the short-range connections in these hubs, however, remains largely unknown but has been hypothesized as a means to maintain stable *in situ* information while simultaneously being able to associate distributed information with distant connections (Sepulcre et al., 2010).

Given the above, it is not surprising that hubs with predominant long-range connections (e.g., anterior insula, central operculum, and posterior cingulate gyrus) have long-range ICC values that exhibited negative association with age, whereas those with predominant short-range connections (e.g., fusiform gyrus and anterior cingulate gyrus) have short-range ICC values that were negatively associated with age. Since whole-brain ICC values simply represent the combination of short-range and long-range values, changes in whole-brain ICC values could be a reflection of the changes in connections in either short-range or long-range or both. For example, age-related changes in the anterior insula were observed in both whole-brain and long-range ICC values (Tables 2, 4). From Figure 2, anterior insula is predominantly a long-range hub so that the observed changes in whole-brain ICC values mainly reflected changes in long-range ICC values. In contrast, posterior insula showed changes in both short-range and long-range ICC values and these changes are also being reflected in the whole-brain ICC values (Figures 3a–5a). This could mean that posterior insula’s connection to both primary processing systems (sensorimotor/auditory) and salience/BG, networks that this region has prominent connections (Figure 3b), could be both affected with age.

Additional seed-based connectivity analyses on regions with ICC values showing significant negative association with age showed that most of the identified regions have connectivity maps that strongly overlapped with not just one but multiple large-scale RSNs. The values of the estimated overlap ratio, computed using whole-brain connections, confirmed that the identified regions are mainly connector hubs, considered important in facilitating information transfer among different brain networks. For instance, the connectivity map of the right medial precentral gyrus overlapped with auditory, sensorimotor, ventral default mode, precuneus, as well as salience networks. Regions in the posterior/anterior insula and posterior/central operculum also overlapped with primary processing networks (sensorimotor, auditory, and primary visual) as well as salience network. These regions are referred to as “control-processing” connector hubs since they linked sensory and motor systems to control networks. Among other functions, control-processing hubs may enable goal-directed control of motor function (Gordon et al., 2018). In contrast, regions in the middle frontal gyrus, angular gyrus, and posterior cingulate gyrus with primarily long-range ICC values that showed significant negative association with age have connectivity maps that strongly overlap with the control (salience, executive, and visuospatial) and default mode networks. These regions are classified as “control-default” connector hubs, which may be responsible for regulating internally generated processes associated with the default mode network such as memories, emotional responses, or planning (Gordon et al., 2018). Age-related alterations of the overall connectivity of these regions could impact its efficiency and thereby affect its functions. Moreover, the effect of aging appeared to vary for different types of connector hubs. Specifically, control-processing connector hubs tend to be affected in both its short-range and long-range (and thus whole-brain) ICC values, whereas control-default connector hubs have primarily long-range ICC values that were negatively associated with age.

In terms of the hubs’ association with general cognitive performance, we have identified regions in the visual and sensorimotor networks with whole-brain and distance-dependent ICC values that showed positive relationship with ACE-R total score. Intriguingly, none of the identified regions overlapped with those having ICC values that negatively associated with age except for a region in the left lingual gyrus, which showed positive relationship with ACE-R total score and negative relationship with age. This finding is consistent with our previous results, which indicated that the integrity of the visual and sensorimotor networks is associated with the participants’ general cognitive performance during healthy aging (Bagarinao et al., 2019). In a prospective study of older women, Ward et al. (2018) have also demonstrated that participants with reduced visual function were associated with greater risk of dementia. Physical exercise has also been shown to help improved cognitive function through increased involvement of motor-related networks (Ji et al., 2018). Thus, our finding relating the overall connectivity strength, as quantified by the ICC values, of the primary processing systems to general cognitive performance is consistent with existing literature and provides additional evidence of the importance of the integrity of the primary processing systems during healthy aging for the maintenance of general cognition.

## Conclusion

Healthy aging is associated with whole-brain intrinsic functional connectivity changes even in the absence of neurodegenerative diseases. Using ICC, we have identified a limited number of regions with overall connectivity strength that showed significant negative relationship with age. More importantly, these regions have functional connectivity profiles that significantly overlapped with not just one but multiple large-scale RSNs indicating that these regions are connector hubs. Connector hubs associated with primary processing (sensorimotor, visual, and auditory) and control networks tended to have whole-brain ICC values that exhibited significant negative relationship with age, whereas control-default connector hubs have predominantly long-range ICC values that exhibited significant negative relationship with age. These findings suggest that even healthy aging could negatively impact, albeit in a limited way, the overall connectivity strength of regions critical in facilitating information transfer among different networks.

## Data Availability Statement

The datasets presented in this article are not readily available because of privacy and ethical restrictions. Requests to access the datasets should be directed to GS, sobueg@med.nagoya-u.ac.jp.

## Ethics Statement

The studies involving human participants were reviewed and approved by the Ethics Committee of Nagoya University Graduate School of Medicine. The patients/participants provided their written informed consent to participate in this study.

## Author Contributions

HW, SM, TT, HT, MKa, TW, MKu, MH, HI, SN, NO, and GS contributed to conception and design of the study. SM, DM, KH, KK, NY, RO, KI, MM, TY, AO, SK, MH, and HI were involved in data acquisition, data organization, and data curation. EB, HW, KK, TT, and HT contributed to the methodology, analysis, and interpretation of the data. EB, HW, SM, and GS wrote the draft of the manuscript. All authors reviewed and approved the final version of the manuscript.

## Conflict of Interest

The authors declare that the research was conducted in the absence of any commercial or financial relationships that could be construed as a potential conflict of interest.

## References

[B1] Achard S. Bullmore E. (2007). Efficiency and cost of economical brain functional networks. *PLoS Comput. Biol.* 3:e17. 10.1371/journal.pcbi.0030017 17274684 PMC1794324

[B2] Achard S. Salvador R. Whitcher B. Suckling J. Bullmore E. (2006). A resilient, low-frequency, small-world human brain functional network with highly connected association cortical hubs. *J. Neurosci.* 26 63–72. 10.1523/JNEUROSCI.3874-05.2006 16399673 PMC6674299

[B3] Alexander-Bloch A. F. Vértes P. E. Stidd R. Lalonde F. Clasen L. Rapoport J. (2013). The anatomical distance of functional connections predicts brain network topology in health and schizophrenia. *Cereb. Cortex* 23 127–138. 10.1093/cercor/bhr388 22275481 PMC3513955

[B4] Andrews-Hanna J. R. Snyder A. Z. Vincent J. L. Lustig C. Head D. Raichle M. E. (2007). Disruption of large-scale brain systems in advanced aging. *Neuron* 56 924–935. 10.1016/j.neuron.2007.10.038 18054866 PMC2709284

[B5] Ashburner J. Friston K. J. (2005). Unified segmentation. *Neuroimage* 26 839–851. 10.1016/j.neuroimage.2005.02.018 15955494

[B6] Bagarinao E. Watanabe H. Maesawa S. Mori D. Hara K. Kawabata K. (2018). An unbiased data-driven age-related structural brain parcellation for the identification of intrinsic brain volume changes over the adult lifespan. *Neuroimage* 169 134–144. 10.1016/j.neuroimage.2017.12.014 29225065

[B7] Bagarinao E. Watanabe H. Maesawa S. Mori D. Hara K. Kawabata K. (2019). Reorganization of brain networks and its association with general cognitive performance over the adult lifespan. *Sci. Rep.* 9:11352. 10.1038/s41598-019-47922-x 31388057 PMC6684569

[B8] Bagarinao E. Watanabe H. Maesawa S. Mori D. Hara K. Kawabata K. (2020). Identifying the brain’s connector hubs at the voxel level using functional connectivity overlap ratio. *Neuroimage* 222:117241. 10.1016/j.neuroimage.2020.117241 32798679

[B9] Beckmann C. F. DeLuca M. Devlin J. T. Smith S. M. (2005). Investigations into resting-state connectivity using independent component analysis. *Philos. Trans. R. Soc. B Biol. Sci.* 360 1001–1013. 10.1098/rstb.2005.1634 16087444 PMC1854918

[B10] Betzel R. F. Byrge L. He Y. Goñi J. Zuo X. N. Sporns O. (2014). Changes in structural and functional connectivity among resting-state networks across the human lifespan. *Neuroimage* 102 345–357. 10.1016/j.neuroimage.2014.07.067 25109530

[B11] Biswal B. Yetkin F. Z. Haughton V. M. Hyde J. S. (1995). Functional connectivity in the motor cortex of resting human brain using echo-planar MRI. *Magn. Reson. Med.* 34 537–541. 10.1002/mrm.1910340409 8524021

[B12] Buckner R. L. Sepulcre J. Talukdar T. Krienen F. M. Liu H. Hedden T. (2009). Cortical hubs revealed by intrinsic functional connectivity: mapping, assessment of stability, and relation to Alzheimer’s disease. *J. Neurosci.* 29 1860–1873. 10.1523/JNEUROSCI.5062-08.2009 19211893 PMC2750039

[B13] Bullmore E. Sporns O. (2012). The economy of brain network organization. *Nat. Rev. Neurosci.* 13 336–349. 10.1038/nrn3214 22498897

[B14] Chan M. Y. Park D. C. Savalia N. K. Petersen S. E. Wig G. S. (2014). Decreased segregation of brain systems across the healthy adult lifespan. *Proc. Natl. Acad. Sci. U.S.A.* 111 E4997–E5006. 10.1073/pnas.1415122111 25368199 PMC4246293

[B15] Crossley N. A. Mechelli A. Scott J. Carletti F. Fox P. T. McGuire P. (2014). The hubs of the human connectome are generally implicated in the anatomy of brain disorders. *Brain* 137 2382–2395. 10.1093/brain/awu132 25057133 PMC4107735

[B16] Dai Z. Yan C. Li K. Wang Z. Wang J. Cao M. (2015). Identifying and mapping connectivity patterns of brain network hubs in Alzheimer’s disease. *Cereb. Cortex* 25 3723–3742. 10.1093/cercor/bhu246 25331602

[B17] Fransson P. Skiold B. Horsch S. Nordell A. Blennow M. Lagercrantz H. (2007). Resting-state networks in the infant brain. *Proc. Natl. Acad. Sci. U.S.A.* 104 15531–15536. 10.1073/pnas.0704380104 17878310 PMC2000516

[B18] Geerligs L. Renken R. J. Saliasi E. Maurits N. M. Lorist M. M. (2015). A Brain-wide study of age-related changes in functional connectivity. *Cereb. Cortex* 25 1987–1999. 10.1093/cercor/bhu012 24532319

[B19] Gordon E. M. Lynch C. J. Gratton C. Laumann T. O. Gilmore A. W. Greene D. J. (2018). Three distinct sets of connector hubs integrate human brain function. *Cell Rep.* 24 1687–1695.e4. 10.1016/j.celrep.2018.07.050 30110625 PMC6886580

[B20] Greicius M. (2008). Resting-state functional connectivity in neuropsychiatric disorders. *Curr. Opin. Neurol.* 21 424–430. 10.1097/wco.0b013e328306f2c5 18607202

[B21] Greicius M. D. Krasnow B. Reiss A. L. Menon V. (2003). Functional connectivity in the resting brain: a network analysis of the default mode hypothesis. *Proc. Natl. Acad. Sci. U.S.A.* 100 253–258. 10.1073/pnas.0135058100 12506194 PMC140943

[B22] Greicius M. D. Srivastava G. Reiss A. L. Menon V. (2004). Default-mode network activity distinguishes Alzheimer’s disease from healthy aging: evidence from functional MRI. *Proc. Natl. Acad. Sci. U.S.A.* 101 4637–4642. 10.1073/pnas.0308627101 15070770 PMC384799

[B23] Hacker C. D. Perlmutter J. S. Criswell S. R. Ances B. M. Snyder A. Z. (2012). Resting state functional connectivity of the striatum in Parkinson’s disease. *Brain* 135 3699–3711. 10.1093/brain/aws281 23195207 PMC3525055

[B24] Hampson M. Tokoglu F. Shen X. Scheinost D. Papademetris X. Constable R. T. (2012). Intrinsic Brain connectivity related to age in young and middle aged adults. *PLoS One* 7:e44067. 10.1371/journal.pone.0044067 22984460 PMC3439483

[B25] He Y. Chen Z. J. Evans A. C. (2007). Small-world anatomical networks in the human brain revealed by cortical thickness from MRI. *Cereb. Cortex* 17 2407–2419. 10.1093/cercor/bhl149 17204824

[B26] Ji L. Pearlson G. D. Zhang X. Steffens D. C. Ji X. Guo H. (2018). Physical exercise increases involvement of motor networks as a compensatory mechanism during a cognitively challenging task. *Int. J. Geriatr. Psychiatry* 33 1153–1159. 10.1002/gps.4909 29851152

[B27] Jones D. T. MacHulda M. M. Vemuri P. McDade E. M. Zeng G. Senjem M. L. (2011). Age-related changes in the default mode network are more advanced in Alzheimer disease. *Neurology* 77 1524–1531. 10.1212/WNL.0b013e318233b33d 21975202 PMC3198977

[B28] Kawabata K. Watanabe H. Hara K. Bagarinao E. Yoneyama N. Ogura A. (2018). Distinct manifestation of cognitive deficits associate with different resting-state network disruptions in non-demented patients with Parkinson’s disease. *J. Neurol.* 265 688–700. 10.1007/s00415-018-8755-5 29392456

[B29] Liang X. Zou Q. He Y. Yang Y. (2013). Coupling of functional connectivity and regional cerebral blood flow reveals a physiological basis for network hubs of the human brain. *Proc. Natl. Acad. Sci. U.S.A.* 110 1929–1934. 10.1073/pnas.1214900110 23319644 PMC3562840

[B30] Martuzzi R. Ramani R. Qiu M. Shen X. Papademetris X. Constable R. T. (2011). A whole-brain voxel based measure of intrinsic connectivity contrast reveals local changes in tissue connectivity with anesthetic without a priori assumptions on thresholds or regions of interest. *Neuroimage* 58 1044–1050. 10.1016/j.neuroimage.2011.06.075 21763437 PMC3183817

[B31] Menon V. (2011). Large-scale brain networks and psychopathology: a unifying triple network model. *Trends Cogn. Sci.* 15 483–506. 10.1016/j.tics.2011.08.003 21908230

[B32] Mugler J. P. Brookeman J. R. (1990). Three-dimensional magnetization-prepared rapid gradient-echo imaging (3D MP RAGE). *Magn. Reson. Med.* 15 152–157. 10.1002/mrm.1910150117 2374495

[B33] Ogura A. Watanabe H. Kawabata K. Ohdake R. Tanaka Y. Masuda M. (2019). Semantic deficits in ALS related to right lingual/fusiform gyrus network involvement. *Ebiomedicine* 47 506–517. 10.1016/j.ebiom.2019.08.022 31492562 PMC6796569

[B34] Power J. D. Barnes K. A. Snyder A. Z. Schlaggar B. L. Petersen S. E. (2012). Spurious but systematic correlations in functional connectivity MRI networks arise from subject motion. *Neuroimage* 59 2142–2154. 10.1016/j.neuroimage.2011.10.018 22019881 PMC3254728

[B35] Sala-Llonch R. Bartrés-Faz D. Junqué C. (2015). Reorganization of brain networks in aging: a review of functional connectivity studies. *Front. Psychol.* 6:663. 10.3389/fpsyg.2015.00663 26052298 PMC4439539

[B36] Seeley W. W. Menon V. Schatzberg A. F. Keller J. Glover G. H. Kenna H. (2007). Dissociable intrinsic connectivity networks for salience processing and executive control. *J. Neurosci.* 27 2349–2356. 10.1523/JNEUROSCI.5587-06.2007 17329432 PMC2680293

[B37] Sepulcre J. Liu H. Talukdar T. Martincorena I. Yeo B. T. T. Buckner R. L. (2010). The organization of local and distant functional connectivity in the human brain. *PLoS Comput. Biol.* 6:e1000808. 10.1371/journal.pcbi.1000808 20548945 PMC2883589

[B38] Shirer W. R. Ryali S. Rykhlevskaia E. Menon V. Greicius M. D. (2012). Decoding subject-driven cognitive states with whole-brain connectivity patterns. *Cereb. Cortex* 22 158–165. 10.1093/cercor/bhr099 21616982 PMC3236795

[B39] Smith S. M. Miller K. L. Salimi-Khorshidi G. Webster M. Beckmann C. F. Nichols T. E. (2011). Network modelling methods for FMRI. *Neuroimage* 54 875–891. 10.1016/j.neuroimage.2010.08.063 20817103

[B40] Smyser C. D. Inder T. E. Shimony J. S. Hill J. E. Degnan A. J. Snyder A. Z. (2010). Longitudinal analysis of neural network development in preterm infants. *Cereb. Cortex* 20 2852–2862. 10.1093/cercor/bhq035 20237243 PMC2978240

[B41] Song J. Birn R. M. Boly M. Meier T. B. Nair V. A. Meyerand M. E. (2014). Age-related reorganizational changes in modularity and functional connectivity of human brain networks. *Brain Connect.* 4 662–676. 10.1089/brain.2014.0286 25183440 PMC4238253

[B42] Sporns O. Honey C. J. Kötter R. (2007). Identification and classification of hubs in brain networks. *PLoS One* 2:e1049. 10.1371/journal.pone.0001049 17940613 PMC2013941

[B43] Tomasi D. Volkow N. D. (2012). Aging and functional brain networks. *Mol. Psychiatry* 17 549–558. 10.1038/mp.2011.81 21727896 PMC3193908

[B44] van den Heuvel M. P. Sporns O. (2013). Network hubs in the human brain. *Trends Cogn. Sci.* 17 683–696. 10.1016/j.tics.2013.09.012 24231140

[B45] van den Heuvel M. P. Sporns O. Collin G. Scheewe T. Mandl R. C. W. Cahn W. (2013). Abnormal rich club organization and functional brain dynamics in schizophrenia. *JAMA Psychiatry* 70 783–792. 10.1001/jamapsychiatry.2013.1328 23739835

[B46] Ward M. E. Gelfand J. M. Lui L.-Y. Ou Y. Green A. J. Stone K. (2018). Reduced contrast sensitivity among older women is associated with increased risk of cognitive impairment. *Ann. Neurol.* 83 730–738. 10.1002/ana.25196 29518257 PMC5947874

[B47] Xia M. Wang J. He Y. (2013). BrainNet viewer: a network visualization tool for human brain connectomics. *PLoS One* 8:e68910. 10.1371/journal.pone.0068910 23861951 PMC3701683

[B48] Yao N. Shek-Kwan Chang R. Cheung C. Pang S. Lau K. K. Suckling J. (2014). The default mode network is disrupted in parkinson’s disease with visual hallucinations. *Hum. Brain Mapp.* 35 5658–5666. 10.1002/hbm.22577 24985056 PMC4657500

[B49] Yokoi T. Watanabe H. Yamaguchi H. Bagarinao E. Masuda M. Imai K. (2018). Involvement of the Precuneus/posterior cingulate cortex is significant for the development of Alzheimer’s disease: a PET (THK5351, PiB) and resting fMRI study. *Front. Aging Neurosci.* 10:304. 10.3389/fnagi.2018.00304 30344488 PMC6182068

[B50] Yoneyama N. Watanabe H. Kawabata K. Bagarinao E. Hara K. Tsuboi T. (2018). Severe hyposmia and aberrant functional connectivity in cognitively normal Parkinson’s disease. *PLoS One* 13:e0190072. 10.1371/journal.pone.0190072 29304050 PMC5755765

