# Supplementary material for: Aging Impacts the Overall Connectivity Strength of Regions Critical for Information Transfer Among Brain Networks
Source: Front Aging Neurosci. 2020 Oct 28;12:592469. doi: 10.3389/fnagi.2020.592469 (PMC7655963; doi:10.3389/fnagi.2020.592469)
Supplement: Supplementary file 1 [file Data_Sheet_1.docx]

## Supplementary Information

**Supplementary Table S1**. Regions showing significant (FWE p < 0.05, cluster size ≥ 25 voxels) whole-brain ICC values using a one-sample t-test of ICC maps in a subgroup of young adult participants.

| Contrast | X | Y | Z | z-Value | Cluster Size | Area | Other Peaks |
| --- | --- | --- | --- | --- | --- | --- | --- |
| Positive | -36 | 22 | 56 | Inf | 10804 | L MFG | L SFG, R MFG |
|  | -18 | -56 | -4 | Inf | 5449 | L LiG | R SOG |
|  | -36 | -66 | 52 | Inf | 3324 | L AnG |  |
|  | 14 | -88 | -30 | 7.17 | 1371 | R Cer |  |
|  | 60 | -16 | -26 | 7.16 | 937 | R MTG | R ITG |
|  | -60 | -32 | -20 | 6.71 | 763 | L ITG | L STG |
|  | -60 | -26 | 18 | 6.56 | 527 | L PO | L CO |
|  | -44 | 34 | -14 | 6.04 | 58 | L LOrG |  |
|  | 0 | 50 | 8 | 5.93 | 61 | R MSFG |  |
|  | 8 | -36 | 52 | 5.85 | 56 | R PCu | R MPoG |
|  | 0 | 48 | -18 | 5.82 | 124 | L GRe | L MFC |
|  | 54 | 16 | 24 | 5.57 | 25 | R OpIFG | R MFG |
|  | -32 | -66 | -28 | 5.49 | 26 | L Cer |  |
|  | -32 | -84 | -4 | 5.49 | 25 | L IOG |  |

MFG, middle frontal gyrus; SFG, superior frontal gyrus; LiG, lingual gyrus; SOG, superior occipital gyrus; AnG, angular gyrus; Cer, cerebellum; MTG, middle temporal gyrus; ITG, inferior temporal gyrus; STG, superior temporal gyrus; PO, posterior operculum; CO, central operculum; LOrG, lateral orbital gyrus; MSFG, medial superior frontal gyrus; PCu, precuneus; MPoG, medial postcentral gyrus; GRe, gyrus rectus; MFC, medial frontal cortex; OpIFG, opercular part of the inferior frontal gyrus; IOG, inferior occipital gyrus; L, left; R, right.

**Supplementary Table S2**. Regions showing significant (FWE p < 0.05, cluster size ≥ 25 voxels) short-range ICC values using a one-sample t-test of ICC maps in a subgroup of young adult participants.

| Contrast | X | Y | Z | z-Value | Cluster Size | Area | Other Within Cluster Peaks |
| --- | --- | --- | --- | --- | --- | --- | --- |
| Positive | 14 | -86 | 32 | Inf | 34212 | R SOG | L Cun |
|  | 0 | 50 | -18 | 7.09 | 510 | L GRe | L MFC, R MSFG |
|  | -36 | -78 | -44 | 6.93 | 1469 | L Cer | R Cer |
|  | 40 | 56 | -12 | 6.28 | 142 | R LOrG |  |
|  | -46 | 32 | -12 | 6.18 | 148 | L OrIFG | L LOrG |
|  | 56 | 8 | 4 | 5.88 | 182 | R OpIFG | R CO |
|  | 58 | -18 | 18 | 5.7 | 47 | R CO |  |
|  | 62 | -26 | 28 | 5.57 | 42 | R SMG |  |

SOG, superior occipital gyrus; Cun, cuneus; GRe, gyrus rectus; MFC, medial frontal cortex; MSFG, medial superior frontal gyrus; Cer, cerebellum; LOrG, lateral orbital gyrus; OrIFG, orbital part of the inferior frontal gyrus; OpIFG, opercular part of the inferior frontal gyrus; CO, central operculum; SMG, supramarginal gyrus; L, left; R, right.

**Supplementary Table S3**. Regions showing significant (FWE p < 0.05, cluster size ≥ 25 voxels) long-range ICC values using a one-sample t-test of ICC maps in a subgroup of young adult participants.

| Contrast | X | Y | Z | z-Value | Cluster Size | Area | Other Peaks |
| --- | --- | --- | --- | --- | --- | --- | --- |
| Positive | -34 | 24 | 58 | Inf | 6120 | L MFG | L SFG, R MFG |
|  | 44 | -62 | 52 | Inf | 4741 | R AnG |  |
|  | 60 | -34 | -18 | Inf | 2478 | R ITG | R MTG |
|  | -36 | -68 | 54 | Inf | 5003 | L AnG |  |
|  | -60 | -30 | -22 | Inf | 2043 | L ITG | L MTG, L STG |
|  | 42 | -72 | -42 | Inf | 1050 | R Cer |  |
|  | 0 | -52 | 30 | 7.69 | 826 | L PCu | L PCgG |
|  | -36 | -74 | -44 | 7.18 | 884 | L Cer |  |
|  | -44 | 34 | -14 | 6.54 | 100 | L LOrG |  |
|  | 56 | 16 | 22 | 5.96 | 72 | R OpIFG | R MFG |
|  | 44 | 38 | -14 | 5.63 | 62 | R LOrG | R OrIFG |
|  | -44 | 46 | 6 | 5.43 | 40 | L MFG |  |

MFG, middle frontal gyrus; SFG, superior frontal gyrus; AnG, angular gyrus; ITG, inferior temporal gyrus; MTG, middle temporal gyrus; STG, superior temporal gyrus; Cer, cerebellum; PCu, precuneus; PCgG, posterior cingulate gyrus; LOrG, lateral orbital gyrus; OpIFG, opercular part of the inferior frontal gyrus; OrIFG, orbital part of the inferior frontal gyrus; L, left; R, right.
